# Effect of microrna-138 on epithelial-Mesenchymal transition and invasion of breast cancer cells by targeting semaphorin 4C

**DOI:** 10.1080/21655979.2021.2000733

**Published:** 2021-12-07

**Authors:** HuiJuan Liu, Hui Ye, Xinzheng Li

**Affiliations:** a Second Ward of Breast Surgery, Shanxi Cancer Hospital, TaiYuan, China; b Third Ward of Thoracic Surgery, Shanxi Cancer Hospital, TaiYuan, China

**Keywords:** Breast cancer, miR-138, semaphorin 4C, epithelial mesenchymal transition, invasion

## Abstract

In view of the role of miR-138 in cancer cells, we predicted the target of miR-138 and its targeting to SEMA4C by bioinformatics software and luciferase experiment. The expression levels of miR-138 in human normal breast epithelial cells and two kinds of BC cells were compared, and the transfection cells were selected. MiR-138 mimetic negative control (miR-NC), miR-138 mimic and miR-138 inhibitor were designed for cell transfection. The results showed that the expression level of miR-138 in MCF-7 cells was the lowest. The up regulation of miR-138 would lead to the high expression of E-cad and the low expression of N-cad, vim and SEMA4C, and the vitality and invasion of BC cells would decrease. The down regulation of miR-138 would lead to the low expression of E-cad and the high expression of N-cad, vim and SEMA4C, and the vitality and invasion of BC cells would increase. miR-138 targeted regulation of SEMA4C can promote the expression of N-cad, inhibit the expression of E-cad, vim and SEMA4C, reverse the EMT of BC cells, and inhibit the activity and invasion of BC cells. MiR-138 has clinical potential as a tumor marker of BC.

## Introduction

Breast cancer (BC) is one of the three major malignant tumors in women. According to statistics, in 2018, the number of newly diagnosed BC patients worldwide reached more than two million, accounting for 11.6% of the total number of new tumors, and more than 600,000 women died of BC, accounting for 6.6% of the total cancer-related deaths [1,2]. At present, the treatment methods of BC mainly include surgical treatment, radiotherapy and chemotherapy, endocrine therapy, etc. However, with the deepening of the understanding of the mechanism of BC occurrence and development and the advancement of molecular biology technology, the application of BC targeted therapy has become more and more extensive [3,4].

MicroRNAs (miRNAs) are small single-stranded RNAs with a size of about 21–23 bases. They are produced from a single-stranded RNA precursor with a hairpin structure of about 70–90 bases after being processed by Dicer. Different from siRNA (double stranded) but closely related to siRNA. It is speculated that these small non-coding RNAs (miRNAs) are involved in regulating gene expression and are closely related to tumor occurrence, development, metastasis and prognosis. Studies have reported that miR-190, miR-138, miR-30a, miR-503-3p and other miRNAs can affect BC cells through target genes. Behavior has an impact [5–7]. Semaphorin (SEMA) 4 C is a secreted cell membrane protein and a member of the semaphorin family. It is up-regulated in various cancers and participates in tumor growth, metastasis and apoptosis through a variety of signal transduction pathways, and cancer progression It is closely related to development. Studies have reported that it is related to the epithelial mesenchymal transition (EMT) of tumor cells [8,9]. EMT means that epithelial cells lose their polarity and tight junctions and adhesion connections between cells under the action of some factors, and become cells with the morphology and characteristics of mesenchymal cells. This behavior will enhance tumor cells’ anti-apoptosis and invasion capabilities [10,11]. According to bioinformatics, it was found that miR-138 and SEMA4C have predicted binding sites, and studies [12] have shown that miR-139 targeting SEMA4C can affect the epithelial-mesenchymal transition of non-small cell lung cancer cells.

Therefore, this study aims to explore the effects of miR-138 targeting SEMA4C in BC cells on epithelial-mesenchymal transition and invasion, to verify the potential of miR-138 as a clinical BC tumor marker, and to provide molecular targeted therapy for breast cancer New reference path.

## Materials and methods

### Materials and Reagents

Human normal breast epithelial cells MCF-10A and BC cells MCF-7 and MDA-MB-231 were purchased from the Shanghai Chinese Academy of Sciences Cell Bank.miR-138 mimic negative control (miR-NC), miR-138 mimic (miR-138 mimic), miR-138 inhibitor (miR-138 inhibitor), miR-138 and U6 primers were all purchased from Shanghai Jima Pharmaceutical Technology Co., Ltd. The wild-type (WT) and mutant (MUT) SEMA4C plasmids were synthesized by Shanghai Shenggong Biological Company; SEMA4C, E-cadherin (E-cadherin), N-cadherin (N-cadhxexrin), vimentin (Vimentin, Vim) primary antibody and corresponding secondary antibody, Transwell chamber were purchased from sigma company in the United States. Trizol RNA extraction kit, reverse transcription kit, Lipofectamine ^TM^ 3000, and MTT kit were all purchased from Thermo Fisher Company, USA; luciferase detection kits were all purchased from Invitrogen Company, USA.

### Cell culture

The MCF-10A, MCF-7, MDA-MB-231 cells were taken out of the liquid nitrogen tank, thawed in a 37°C water bath, and inoculated on DMEM medium (Gibco, USA) containing 10% fetal bovine serum (FBS), and placed Culture in a constant temperature incubator at 37°C, 95% humidity, 5% CO_2_, and changed the medium regularly.

### Q-PCR

MCF-10A, MCF-7, and MDA-MB-231 cells in logarithmic growth phase were used to extract total RNA by the Trizol method, and the RNA was reverse transcribed into cDNA according to the instructions of the reverse transcription kit. qRT-PCR was used for quantitative PCR amplification. The primer sequences of miR-138 and U6 are shown in Table 1. The total reaction system was 20 μL, containing 2xSYBR MIX 10 μL, Taq II DNA polymerase 0.2 μL, miR-138 or U6 reverse transcription product 2.0 μL, double distilled water (ddH2O) 7.4 μL, 5 μmol/L miR-138 or U6 Specific primers.reaction conditions: pre-denaturation at 95°C for 3 minutes, followed by 40 cycles of denaturation at 95°C for 12s, annealing at 62°C for 35s, and extension at 72°C for 30s. The CT value was subsequently calculated. The relative expression of miR-138 is calculated with 2^−ΔΔCT^ [13,14], ΔΔCT = ΔCT _experimental group_-ΔCT _control group_; ΔCT _experimental group_ = CT _target gene, experimental group_-CT _internal reference gene, experimental group_; ΔCT _control group_ = CT _target gene, control group_-CT _internal reference gene, control group_.

**Table 1.** Primer sequence

name	project	Sequence (5ʹ-3ʹ)
miR-138	Reverse transcription primer	GTCGTATCCAGTGCAGGGTCCGAGGTATTCGCACTGGATACGACTCGACCA
	Forward primer	TCCGAGCCTGACTAAGTGTTGTGGTCGA
	Reverse primer	GTGCAGGGTCCGAGGT
U6	Reverse transcription primer	GTCGTATCCAGTGCAGGGTCCGAGGTATTCGCACTGGATACGACAAAATA
	Forward primer	TCCGATCGTGAAGCGTTC
	Reverse primer	GTGCAGGGTCCGAGGT

### Cell transfection

MCF-10A cells in the logarithmic growth phase were seeded on a 6-well culture plate. When the cells grew to a confluency of more than 80%, the transfection followed the instructions of the Lipofectamine ^TM^ 3000 kit to transfer miR-NC, miR-138 mimic and miR-138 inhibitor were transfected into MCF-10A cells, respectively, and cultured in a constant temperature incubator at 37°C, 95% humidity, and 5% CO_2_.

### Western blot detected the expression of EMT-related proteins and SEMA4C

After the cells were transfected for 48 hours, the cells were lysed with RIPA lysis buffer, and the total proteins of the transfected cells in each group were collected. After 2% polyacrylamide gel electrophoresis (SDS-PAGE), polyvinylidene fluoride (PVDF) was transferred to the membrane, TBST solution was blocked for 90 minutes, and E-cadherin (E-cad), N-cadherin (N-cad), Vimentin (Vim), and SEMA4C antibodies were added (1: 1000 dilution), incubate overnight at 4°C. Horseradish peroxidase (HRP)-labeled secondary antibody (diluted 1:1000) was added in the next day, and incubated for 1 hour at room temperature for color development. Using β-actin as an internal reference, Quantity One software analyzed the gray value of the band, and the ratio of the gray value of protein to β-actin was used as the relative protein expression. The experiment was repeated 3 times and the average was taken.

### MTT test detected cell viability

After 48 hours of transfection, the cells of each group were seeded in a 96-well plate with a cell concentration of 2 × 10^3^ cells/well, each group had 6 replicates, and placed in a 37°C, 5% CO_2_ constant temperature incubator for 12 hours, 24 hours, 48 h, 72 h. 20 μL of MTT solution was added at a time according to the time point, and the culture was continued for 4 hours. The absorbance (OD) was measured at 490 nm. The experiment was repeated 3 times and the average was taken.

### Transwell chamber experiment detected cell invasion

Cells of each group in the logarithmic growth phase after transfection were added to Matrigel glue to coat the Transwell chamber at 10^4^ cells/well, and placed in a 37°C, 95% humidity, 5% CO_2_ constant temperature incubator for 24 hours. The cells and excess Matrigel glue in the upper chamber were gently wiped with a cotton swab, fixed with 4% formaldehyde for 20 minutes, washed with PBS 3 times, and stained with 0.1% crystal violet for 15 minutes. 5 visual fields under the microscope were randomly selected to observe the number of invaded cells after washing with PBS. The experiment was repeated 3 times and the average was taken.

### Luciferase experiment

According to the complementary sequence of miR-138 and SEMA4C predicted by the bioinformatics software Targetscan, the fragment of the binding site was amplified and the point mutation technique was used to construct WT-SEMA4C and MUT-SEMA4C plasmids. WT-SEMA4C, MUT-SEMA4C, miR-NC, miR-138 mimic were co-transfected into MCF-7 cells, and the fluorescence intensity was measured after 48 h. The firefly luciferase was used as the internal control, and Renilla luciferase was used for the determination. The relative luciferase activity was the ratio of firefly luciferase to Renilla luciferase. The experiment was repeated 3 times and the average was taken.

### Statistical Methods

SDS1.4 software was used to calculate the sample CT value. The SPSS21.0 software was used for processing, and the count data was expressed by frequency (n), and χ^2^ test was used. Measurement data were expressed as mean ± standard deviation, $\bar{(x}\;\pm s)$ and t test was used for pairwise comparison between groups. The test level was α = 0.05, and *P*< 0.05 indicates that the difference was statistically significant.

## Results and discussion

### Detection of miR-138 and SEMA4C gene binding sites

**Figure 1.**
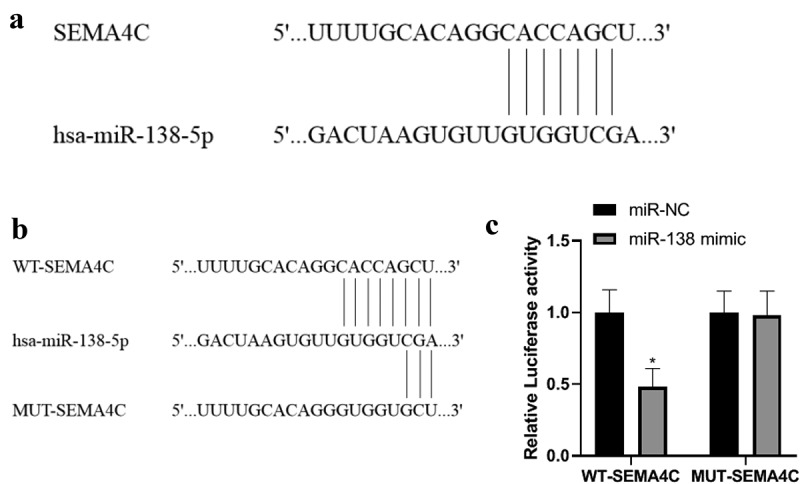
Detection of miR-138 and SEMA4C gene binding sites. Note: (a) Results of bioinformatics software for miR-138 and SEMA4C gene binding sites; (b) Construction of WT-SEMA4C and MUT-SEMA4C; (c) miR-138 targeted regulation SEMA4 luciferase experimental results; *P< 0.05, compared with the miR-NC group

In order to explore the relationship between miR-138 and SEMA4C, we carried out miR-138 and SEMA4C gene binding site detection and dual luciferase experiments. The bioinformatics software Targetscan predicted that miR-138 has a binding site fragment with SEMA4C. As shown in Figure 1a. The luciferase experiments showed that in the cells co-transfected with miR-138 and WT-SEMA4C, the relative luciferase activity was reduced (*P*< 0.05), while in the cells co-transfected with miR-NC and MUT-SEMA4C, The relative luciferase activity did not change (*P*> 0.05), indicating that SEMA4C is the target gene of miR-138. As shown in Figure 1(b-c).

### Comparison of miR-138 expression in BC cells

**Figure 2.**
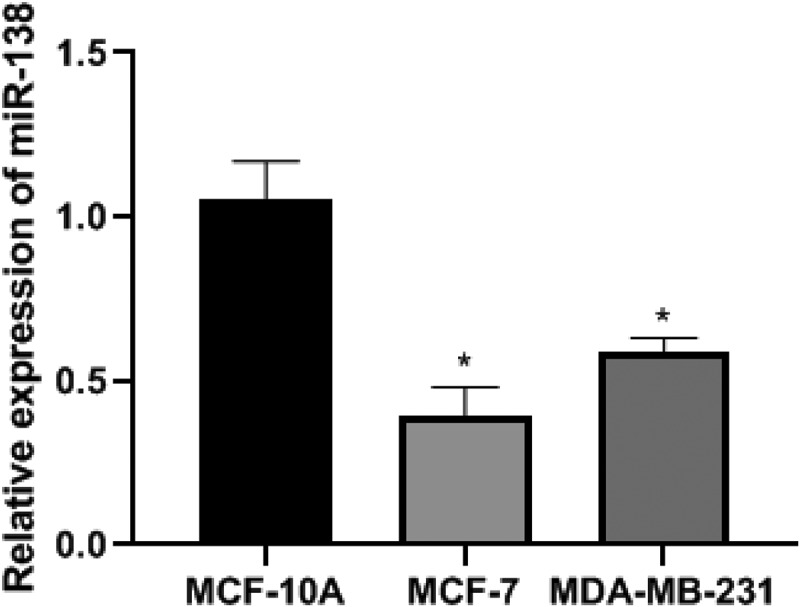
The expression of miR-138 in BC cells of each group. Note: *P< 0.05, compared with the MCF-10A group

In order to detect the expression of miR-138 in BC cells, we conducted PCR experiments and conducted cell screening. The PCR results showed that the relative expression levels of miR-138 in MCF-10A, MCF-7 and MDA-MB-231 cells were 1.05 ± 0.12, 0.39 ± 0.09, 0.59 ± 0.04, respectively.The relative expression levels of miR-138 in MCF-7 and MDA-MB-231 cells were lower than MCF-10A. The differences were statistically significant (*P*< 0.05). Among them, the relative expression level of miR-138 in MCF-7 cells was the lowest, so MCF-7 cells were selected as cells for transfection. The differences were statistically significant (*P*< 0.05). As shown in Figure 2.

### Expression of miR-138, EMT-related protein and SEMA4C protein after transfection

**Figure 3.**
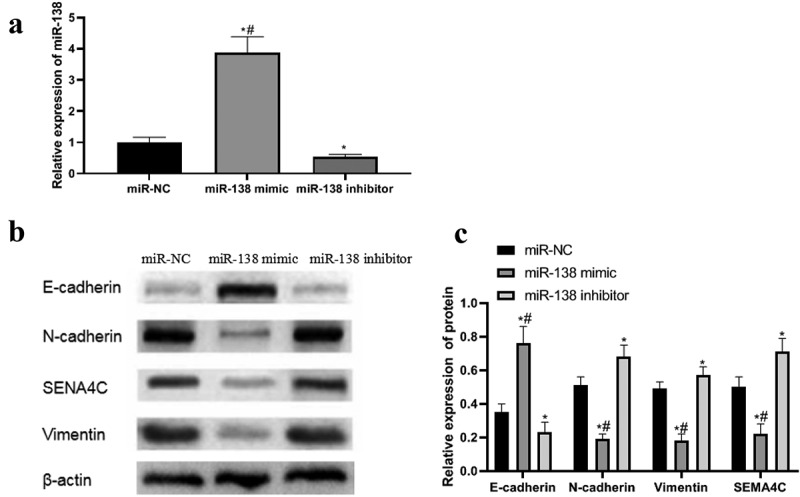
PCR and western blot results. Note: (a) Expression of miR-138 in transfected cells; (b) Western blot to detect the effects of miR-138 mimic and miR-138 inhibitor on the expression of SEMA4C and EMT-related proteins in cells; (c) Relative protein expression of SEMA4C and EMT-related proteins in transfected cells; *P< 0.05, compared with the miR-NC group; ^#^*P*< 0.05, compared with the miR-183 inhibitor group

In order to detect the success of the transfection and explore the effect of miR-138 on the expression of EMT and SEMA4C, we performed PCR and Western blot experiments to detect the expression of miR-138, EMT-related proteins and SEMA4C in the cells after transfection. PCR and Western blot results showed that the expression levels of miR-138 and E-cad in the miR-138 mimic group were higher than those in the miR-NC group and miR-138 inhibitor group, while the expression levels of N-cad, Vim and SEMA4C were lower than those in the miR-NC group And miR-138 inhibitor group, The differences were statistically significant (*P*< 0.05). The expression levels of miR-138 and E-cad in the miR-138 inhibitor group were lower than those in the miR-NC group, while the expression levels of N-cad, Vim and SEMA4C were higher than those in the miR-NC group. The differences were statistically significant (*P*< 0.05). As shown in Figure 3(a-c).

### miR-138 targets and regulates SEMA4C to inhibit cell viability

**Figure 4.**
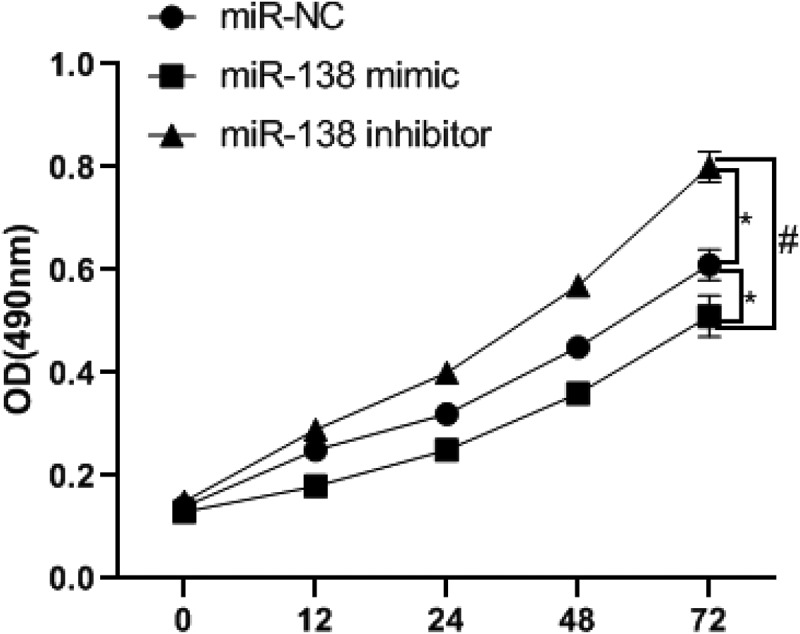
MiR-138 targets SEMA4 to inhibit cell proliferation. Note: *P< 0.05, compared with the miR-NC group; ^#^*P*< 0.05, compared with the miR-183 inhibitor group

In order to detect the effect of miR-138 regulating SEMA4C on cell viability, we used MTT experiment to detect the absorbance of cells at 0, 12, 24, 48, 72 h. The results of the MTT experiment showed that the cell viability of the miR-138 mimic group was lower than that of the miR-NC group and miR-138 inhibitor group from the 12th hour, The differences were statistically significant (*P*< 0.05). Since the 24th hour, the cell viability of miR-138 inhibitor group was higher than that of miR-NC group. The differences were statistically significant (*P*< 0.05). As shown in Figure 4.

### miR-138 targets SEMA4C to inhibit cell invasion

**Figure 5.**
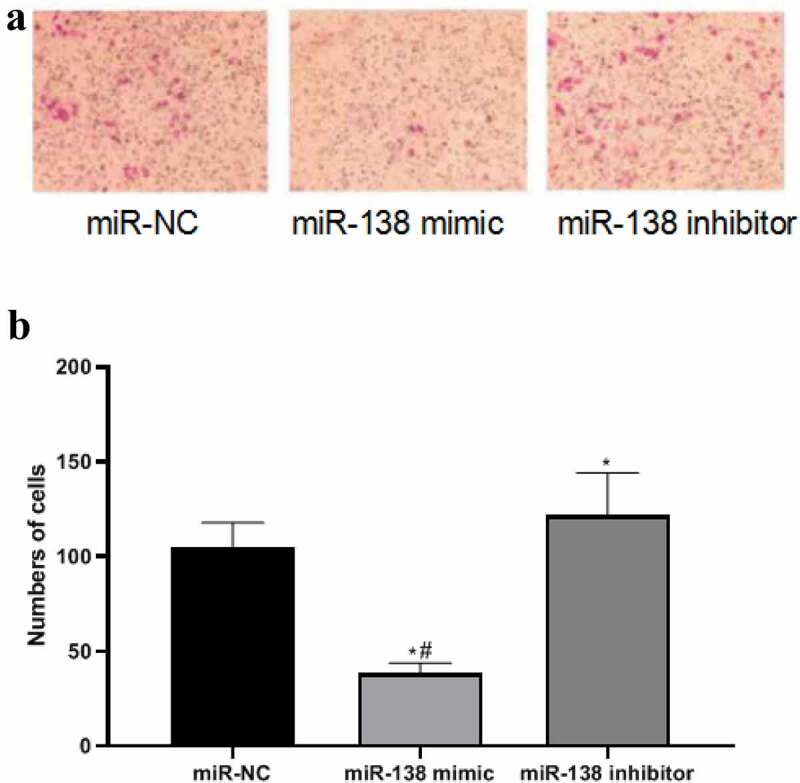
MiR-138 targets SEMA4C to inhibit cell invasion. Note: (a) Transwell detects the effects of miR-138 mimic and miR-138 inhibitor on cell invasion (200×); (b) Comparison of the number of cells invaded by transfected cells; *P< 0.05, compared with the miR-NC group; ^#^*P*< 0.05, compared with the miR-183 inhibitor group

In order to detect the effect of miR-138 regulating SEMA4C on cell invasion, we used the Transwell chamber experiment to detect the number of cells invaded after transfection. The results of the Transwell chamber experiment showed that the number of cell invasions in the miR-NC group, miR-138 mimic group and miR-138 inhibitor group were 104.72 ± 13.22, 38.85 ± 5.05, and 131.81 ± 22.43, respectively. The number of invasive cells in miR-138 mimic group was lower than that of miR-NC group and miR-138 inhibitor group, The differences were statistically significant (*P*< 0.05). The number of invasive cells in the miR-138 inhibitor group was higher than that in the miR-NC group, The differences were statistically significant (*P*< 0.05). As shown in Figure 5(a-b).

## Discussion

BC is a phenomenon in which breast epithelial cells proliferate out of control under the action of a variety of carcinogens. The early stage of the disease often manifests as breast lumps, nipple discharge, axillary lymphadenopathy and other symptoms. In the late stage, cancer cells may metastasize and multicellular disease may appear, which directly threatens the life of the patient. BC is the highest incidence of malignant tumors among Chinese women, and the incidence is increasing year by year, which seriously threatens the lives and health of Chinese women [15]. The etiology of BC is not clear. So far, scientists have not found the exact cause of carcinogenesis of BC, but many high-risk factors related to the onset of BC have been found. In order to better diagnose and treat BC, it is necessary to study the mechanism of its occurrence and development.

EMT refers to the biological process of epithelial cells transforming into cells with mesenchymal phenotype through specific procedures. It plays an important role in embryonic development, chronic inflammation, tissue remodeling, cancer metastasis and various fibrotic diseases. Its main characteristics are the reduction of cell adhesion molecule expression. And the conversion of cytokeratin cytoskeleton to vimentin-based cytoskeleton and the morphology has the characteristics of mesenchymal cells. Through EMT, epithelial cells lost their cell polarity, lost their connection with basement membrane and other epithelial phenotypes, and obtained higher mesenchymal phenotypes such as migration and invasion, anti apoptosis and degradation of extracellular matrix. EMT is a cell biological process related to tumor cell’s anti-apoptosis, invasion and metastasis. During EMT, the expression of E-cad decreases and the cell adhesion ability decreases. The tumor metastasis is enhanced, and the high expression of N-cad and Vim promotes the transformation of cells to mesenchyme and promotes the proliferation of blood vessels, which improves the invasion ability of the tumor [16,17].

miRNA is a type of endogenous small RNA with a length of about 20–24 nucleotides, which has a variety of important regulatory effects in cells. Each miRNA can have multiple target genes, and several miRNAs can also regulate the same gene. This complex regulatory network can not only regulate the expression of multiple genes through one miRNA, but also fine-tune the expression of a certain gene through the combination of several miRNAs. The miR-183 family contains three members: miR-183, miR-96 and miR-182, which are located on the human 7q32.2 chromosome and are transcribed in the same direction [18]. According to investigations, members of the miR-183 family are involved in the regulation of a variety of non-tumor and tumors [18–21]. It is worth noting that many studies have shown that miR-183 is expressed in BC and plays a certain regulatory role [22–24].

The semaphorin is a family of secreted and membrane-related proteins involved in axon guidance, cell differentiation, cytoskeletal rearrangement and cell movement. Members of the semaphorin family also regulate tumor cell proliferation, angiogenesis and immune response [25]. SEMA4C is related to the proliferation, metastasis and invasion of a variety of tumor cells, but there is no relevant research on its influence mechanism on BC cells [26]. this study verified the effect of miR-138 targeting SEMA4C on the EMT-related proteins of BC cells to verify that miR-138 targeting SEMA4C acts on the epithelial-mesenchymal transition of BC cells, thereby affecting the viability and metastasis of BC cells.

According to the bioinformatics software Targetscan, the predicted binding site fragments of miR-138 and SEMA4C were obtained, and WT-SEMA4C and MUT-SEMA4C were constructed for luciferase experiments. The results proved that miR-138 and SEMA4C had binding sites, and SEMA4C was The target gene of miR-138 is consistent with the research results of Li et al. [12]. In this study, after comparing the expression levels of miR-138 in human normal breast epithelial cells MCF-10A and BC cells MCF-7 and MDA-MB-231, the MCF-7 cells with the lowest expression levels were selected as transfected cells.

After cell transfection, PCR and Western blot experiments showed that the up-regulation of miR-138 would lead to high expression of E-cad and low expression of N-cad, Vim and SEMA4C, while down-regulation of miR-138 would cause E-cad Low expression and high expression of N-cad, Vim and SEMA4C. It shows that miR-138 targeting inhibition of SEMA4C expression can reverse the EMT of BC cells. The MTT experiment found that miR-138 targeting SEMA4C would inhibit the viability of BC cells. This result may be related to the reversal of the EMT process [27]. In the Transwell chamber experiment, compared with BC cells with high expression of miR-138, BC cells with low expression of miR-138 had stronger invasion ability. According to the research of Liang et al. [28], the expression of E-cad and Vim can enhance cells Invasive ability.

## Conclusion

In summary, SEMA4C is the target gene of miR-138. In BC cells, miR-138 targeted regulation of SEMA4C can promote the expression of N-cad, inhibit the expression of E-cad, Vim, and SEMA4C, and reverse the EMT of BC cells, thereby inhibiting the viability and invasion ability of BC cells. miR-138 has clinical potential as a BC tumor marker. Nevertheless, this study still needs to further explore the effect of miR-138 on other target genes in BC cells to rule out the possible influence of other target genes on the EMT process.
